# A Challenging Case of Wild-Type Transthyretin Amyloidosis (ATTR) Amyloidosis Treated With Cardiac Resynchronization Therapy

**DOI:** 10.7759/cureus.87203

**Published:** 2025-07-02

**Authors:** Hiroki Bansho, Daisuke Tomioka, Toru Geshi, Hiroshi Sakai, Yoshihisa Nakagawa

**Affiliations:** 1 Cardiology, Shiga University of Medical Science, Otsu, JPN; 2 Cardiology, Otsu Red Cross Hospital, Otsu, JPN; 3 Cardiology, Hikone Municipal Hospital, Hikone, JPN

**Keywords:** cardiac resynchronization therapy, heart failure, left bundle branch block, reduced ejection fraction, wild-type attr amyloidosis

## Abstract

Wild-type transthyretin amyloidosis (ATTRwt) is an age-associated systemic disorder characterized by extracellular deposition of misfolded transthyretin amyloid fibrils, leading to progressive organ dysfunction. Cardiac involvement is common and may result in restrictive cardiomyopathy, arrhythmias, and conduction disturbances, including left bundle branch block (LBBB). Although pharmacological therapy with transthyretin stabilizers, such as tafamidis, has been shown to reduce mortality in patients with ATTRwt cardiac amyloidosis (ATTRwt-CA), the role of device-based therapies, such as cardiac resynchronization therapy (CRT), remains controversial, particularly in patients with coexisting conduction abnormalities.

We report the case of an 81-year-old woman diagnosed with ATTRwt-CA who presented with symptomatic heart failure and LBBB. Tafamidis therapy was initiated to improve her exercise tolerance and to reduce her risk of mortality. Owing to the presence of LBBB and evidence of mechanical dyssynchrony, CRT was also introduced in an effort to prevent the deterioration of her heart failure with reduced ejection fraction. Device optimization was guided by gated myocardial perfusion imaging, performed using single photon emission computed tomography (SPECT), which enabled a detailed assessment of her left ventricular synchrony and informed CRT programming.

Following CRT implantation and optimization, the patient exhibited marked symptomatic improvement, including resolution of her exertional dyspnea and fatigue. Objective assessments demonstrated improved left ventricular contractility and reverse remodeling, suggesting a favorable response to CRT. This case underscores the potential value of CRT in selected patients with ATTRwt-CA and conduction system disease, particularly when mechanical dyssynchrony is evident. Furthermore, it highlights the utility of nuclear imaging modalities such as SPECT in guiding CRT optimization in this unique patient population. Prospective studies are warranted to better define the indications, predictors of response, and long-term outcomes of CRT in patients with ATTRwt-CA.

## Introduction

Wild-type transthyretin (TTR) amyloidosis (ATTRwt) is a progressive, age-related systemic disease characterized by the deposition of misfolded TTR protein in the form of insoluble amyloid fibrils in various organs and tissues. Unlike hereditary ATTR amyloidosis, which is caused by mutations in the TTR gene, ATTRwt amyloidosis arises from the inherent instability of the wild-type TTR tetramer. This instability leads to dissociation into monomers, misfolding, and aggregation of amyloid fibrils that are deposited in the extracellular matrix [1]. ATTRwt amyloidosis predominantly affects older adults and has historically been underdiagnosed owing to its nonspecific clinical presentation and overlap with other age-related conditions.

ATTRwt amyloidosis causes organ-specific lesions, most notably in musculoskeletal structures such as the tendons of the carpal tunnel and spinal ligaments, the autonomic nervous system, and the cardiovascular system [2]. Cardiac involvement, commonly referred to as ATTRwt cardiac amyloidosis (ATTRwt-CA), is a major cause of morbidity and mortality. The clinical presentation of ATTRwt-CA typically includes restrictive cardiomyopathy, progressive heart failure with a preserved or mildly reduced ejection fraction (EF), and arrhythmias [3]. Electrocardiographic (ECG) abnormalities, including conduction disturbances such as atrioventricular or intraventricular conduction disorders, are frequently observed and complicate disease management.

Recently, substantial advances have been made in the diagnosis and treatment of ATTRwt-CA. Non-invasive imaging modalities, such as technetium-99m-labeled bone scintigraphy, cardiac magnetic resonance imaging (MRI), and echocardiography with strain analysis, have facilitated earlier and more accurate diagnoses [4]. Disease-modifying pharmacological agents such as tafamidis, which stabilize the TTR tetramer, have demonstrated efficacy in slowing disease progression and improving survival in patients with ATTRwt-CA [5,6]. Despite these advances, the efficacy of further treatment for residual heart failure symptoms and conduction system disease remains unclear.

Cardiac resynchronization therapy (CRT) is an established intervention for patients with heart failure and intraventricular conduction delay, particularly left bundle branch block (LBBB), which contributes to mechanical dyssynchrony and impaired ventricular function [7]. However, the efficacy of CRT in patients with ATTRwt-CA remains unclear, and current guideline-directed therapy offers limited specific recommendations for this unique patient population [8]. Given the diffuse myocardial infiltration, reduced compliance, and atypical remodeling patterns observed in amyloidosis, the response to CRT may be unpredictable.

This report presents a clinically challenging case in which CRT was implemented in a patient with ATTRwt-CA complicated with LBBB. In this case, we sought to optimize the therapy by using nuclear medicine imaging to assess mechanical dyssynchrony and guide device programming. The patient demonstrated early symptomatic improvement and enhanced left ventricular systolic function over the subsequent months. This case highlights the potential utility of CRT in patients with ATTRwt-CA and provides insights into individualized device-based therapy in this complex disease setting.

## Case presentation

The patient was an 81-year-old Japanese woman with a known history of heart failure with reduced EF (HFrEF), previously documented to have a left ventricular EF (LVEF) of 28% (Table 1).

**Table 1:** The patient’s medical history.

Timeline	Events
71 years old	She presented with exertional dyspnea and was diagnosed with heart failure with a reduced ejection fraction (HFrEF; 28%). Coronary angiography revealed no organic stenosis, and late gadolinium-enhancement magnetic resonance imaging revealed no fibrosis. The patient underwent pharmacotherapy for HFrEF due to the left bundle branch block.
80-year-old	She was admitted to Hikone Municipal Hospital with heart failure. She had been experiencing HFrEF (35%). Right ventricular endomyocardial biopsy revealed amyloid deposition in the myocardial tissue. Genetic testing ruled out mutations in the transthyretin (TTR) gene. The patient was diagnosed with wild-type transthyretin cardiac amyloidosis.
81-year-old	She was referred to Shiga University of Medicine for treatment of wild-type transthyretin amyloidosis. Tafamidis and cardiac resynchronization therapy was administered.

**Figure 1:**
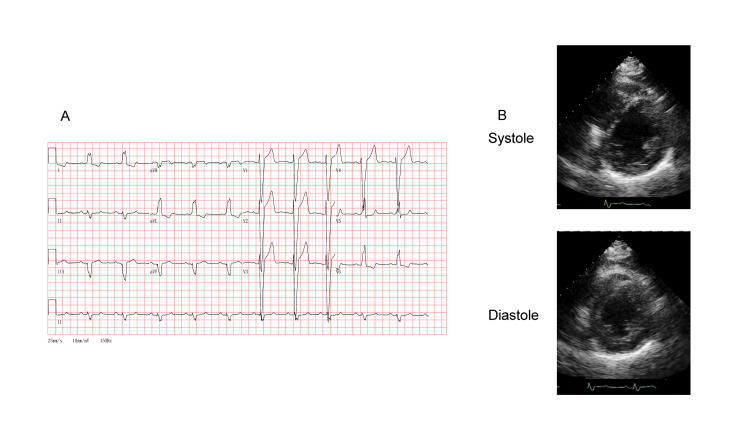
Electrocardiography (ECG) and transthoracic echocardiography (TTE) at the time of admission. (A) ECG revealed a sinus rhythm and left bundle branch block. (B) TTE revealed diffuse left ventricular wall thickening (12 mm) and a reduced ejection fraction. Top: end systole in the short-axis view. Bottom: end diastole in the short-axis view.

She was admitted to the cardiology department because of worsening symptoms of heart failure that were refractory to optimal pharmacological therapy, including diuretics and guideline-directed medical treatment. On admission, the patient’s blood pressure was 132/74 mmHg, and pulse rate was 67 beats per minute. Physical examination revealed regular heart sounds without murmurs, and pulmonary auscultation yielded no remarkable observations and no rales. We observed evidence of musculoskeletal involvement, including thenar eminence atrophy and an inability of the patient to form a positive “O” sign with her right hand. Her physical functional status was classified as New York Heart Association (NYHA) class III. A twelve-lead ECG revealed a sinus rhythm with LBBB and a widened QRS complex (166 ms) (Figure 1A). Transthoracic echocardiography (TTE) demonstrated an increased left ventricular wall thickness (12 mm), reduced LVEF (33%), and elevated end-systolic volume (ESV; 101 mL) (Figure 1B), which was consistent with infiltrative cardiomyopathy and HFrEF.

**Figure 2:**
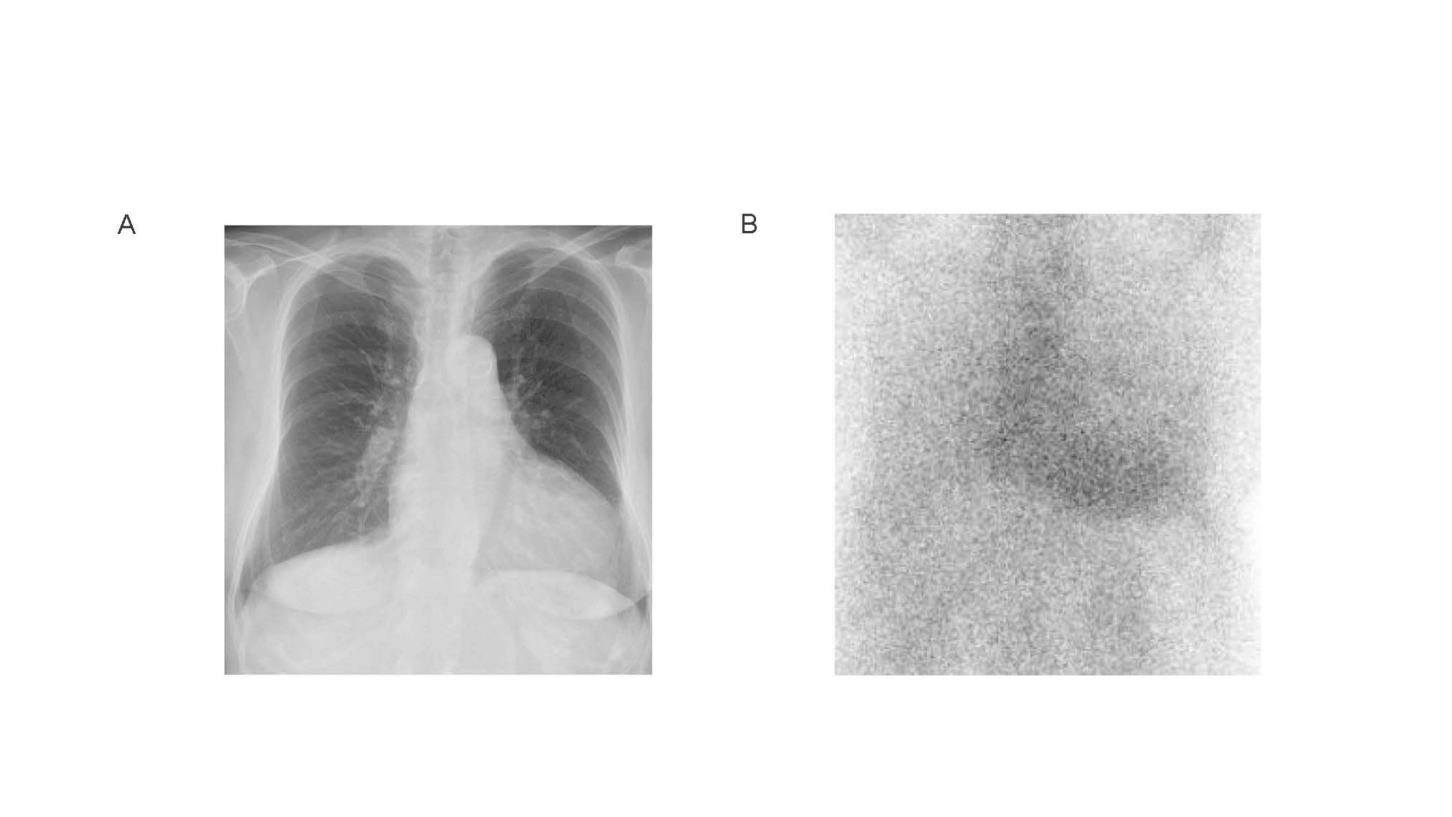
Chest X-ray and pyrophosphate scintigraphy at the time of diagnosis. (A) The cardiothoracic ratio was 58%, indicating cardiac dilation. (B) ^99m^Tc-pyrophosphate scintigraphy revealed a high level (grade 2) of myocardial uptake of radiotracer.

Chest X-ray revealed a cardiothoracic ratio of 58% (Figure 2A), and 99mTc-pyrophosphate scintigraphy revealed a high level of myocardial uptake of radiotracer (Figure 2B).

**Figure 3:**
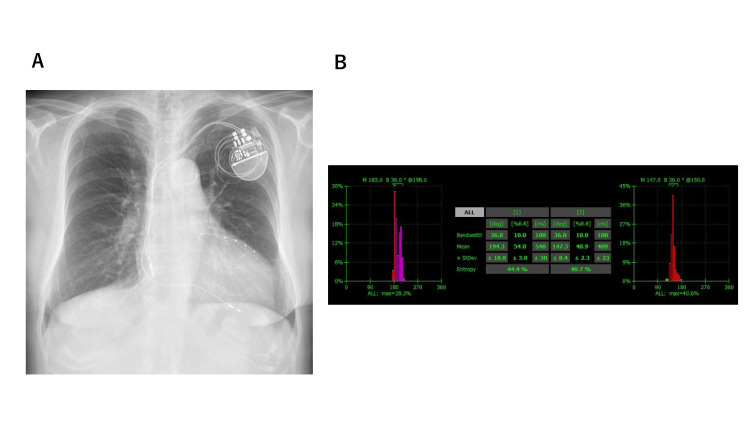
Chest X-ray and single photon emission computed tomography (SPECT) after implantation of the cardiac resynchronization therapy device. (A) The X-ray revealed the ventricular lead in the right ventricle and coronary vein. (B) Quantitative gated SPECT revealed the phase standard deviation (SD) variability and histogram bandwidth (BW). Left: biventricular pacing. Right: left ventricular (LV) pacing. LV pacing aligned more closely with the normal range for SD and BW.

Laboratory investigations revealed a serum B-type natriuretic peptide (BNP) level of 96 ng/mL and a high-sensitivity troponin I level of 32 ng/mL. In accordance with the diagnostic and therapeutic guidelines of the Japanese Circulation Society for TTR amyloidosis, treatment with tafamidis was initiated to stabilize the TTR tetramer and to prevent further amyloid fibril formation and cardiac dysfunction. Given the presence of LBBB and HFrEF, CRT was initiated to improve cardiac synchrony and reverse myocardial remodeling (Figure 3A). To guide and optimize CRT settings, myocardial perfusion single photon emission computed tomography (SPECT) was performed. Quantitative gated SPECT (QGS; Cedars-Sinai Medical Center, Los Angeles, CA, USA) was used to assess mechanical dyssynchrony by analyzing phase standard deviation (SD) and histogram bandwidth (BW). The SPECT results identified left ventricle-only pacing as the most favorable strategy, as it was most closely aligned with the normal SD and BW values (Figure 3B).

**Figure 4:**
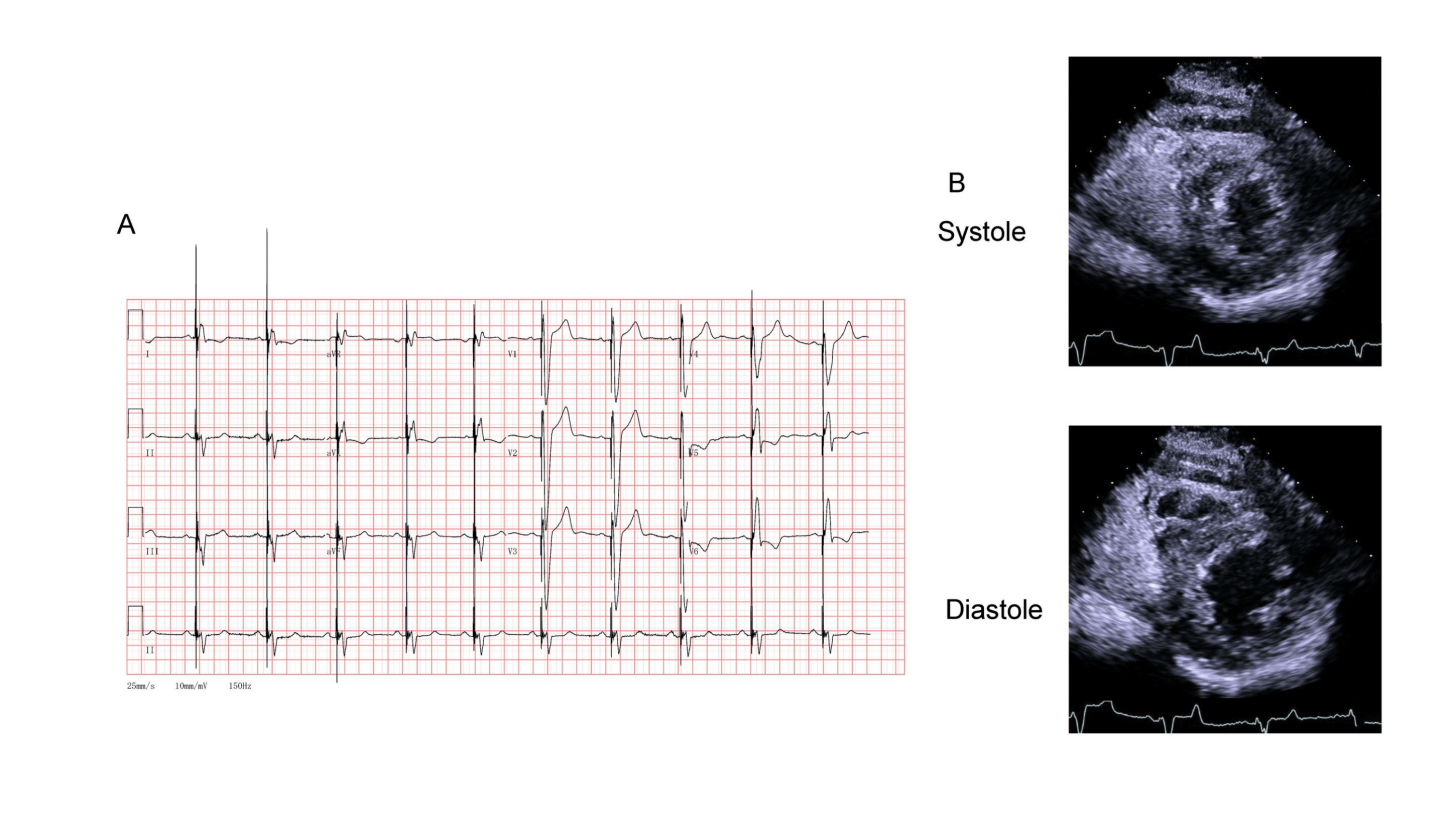
Electrocardiography (ECG) and transthoracic echocardiography (TTE) 2 months after cardiac resynchronization therapy (CRT). (A) ECG revealed a pacemaker rhythm. (B) TTE. Top: end systole in the short-axis view. Bottom: end diastole in the short-axis view.

By two months after CRT device implantation and optimization, the patient's clinical and imaging characteristics had significantly improved. ECG revealed narrowing of the QRS complex to 144 ms (Figure 4A). TTE revealed an improved LVEF (47%) and reduction in the ESV to 59 mL (Figure 4B).

Concurrently, the BNP level had decreased to 25 ng/mL, reflecting an improvement in the patient’s hemodynamic status. Follow-up at eight months demonstrated a sustained therapeutic benefit. TTE revealed a maintained LVEF (45%) and ESV (60 mL); the BNP level remained relatively stable (31 ng/mL), and the high-sensitivity troponin I level was 30 ng/mL. The patient also reported an improvement in exercise tolerance and quality of life, with a reduction in heart failure symptoms. Her NYHA classification was class II.

## Discussion

This case highlights the potential utility of CRT in a patient with ATTRwt-CA, a condition characterized by progressive myocardial infiltration of misfolded TTR fibrils that results in restrictive cardiomyopathy and conduction system disease. Although pharmacological management with tafamidis has been shown to significantly reduce all-cause mortality and delay disease progression when initiated early [3,6], its mechanism, stabilizing the TTR tetramer, does not involve degradation or removal of existing amyloid fibrils. Improvements in parameters such as EF have not been reported for tafamidis therapy alone.

CRT is a well-established intervention in patients with heart failure and electrical dyssynchrony, particularly those with an EF <35% and LBBB, demonstrating reductions in symptoms and improvements in the EF and survival [8-10]. However, its role in conduction abnormalities, which characterize this disease, remains unclear [11]. In this challenging case, CRT resulted in a notable reduction in heart failure symptoms and recovery of the EF, suggesting a synergistic benefit when combined with disease-stabilizing pharmacotherapeutic agents such as tafamidis. This outcome supports the notion that CRT may offer functional benefits even in the context of an amyloid-laden myocardium, particularly in the presence of LBBB. The selection of candidates with ATTRwt-CA for CRT requires a nuanced and individualized approach. Standard selection criteria, including QRS duration and morphology, the EF, and clinical status, remain relevant but may be insufficient. The efficacy of an implantable cardioverter-defibrillator in improving the patient prognosis also remains unclear [12,13]. After deliberation with our heart failure team, the decision was made to proceed with a CRT pacemaker.

The potential for advanced imaging modalities, such as phase analysis via gated SPECT, to provide objective markers of mechanical dyssynchrony was first demonstrated by Chen et al. [14], who introduced a histogram-based method to assess the phase-angle distribution across the left ventricle. In our case, CRT optimization based on parameters approximating the normal phase-angle dispersion yielded sustained ventricular synchrony and a stable ESV over an eight-month follow-up period [15]. This underscores the importance of individualized device programming and the potential of quantitative imaging biomarkers in therapeutic decision-making. Given the rarity of ATTRwt-CA and its complex pathophysiology, further prospective studies are warranted to validate the use of CRT in this population and explore the utility of SPECT-derived mechanical dyssynchrony indices as potential tools for patient selection and CRT optimization.

## Conclusions

This case highlights the potential efficacy of CRT in selected patients with ATTRwt-CA and other conduction abnormalities. The utility of nuclear imaging techniques such as SPECT for CRT optimization may be particularly valuable in patients with complex cardiomyopathies with diffuse myocardial involvement.
